# Non-farm entrepreneurship in rural sub-Saharan Africa: New empirical evidence

**DOI:** 10.1016/j.foodpol.2016.09.019

**Published:** 2017-02

**Authors:** Paula Nagler, Wim Naudé

**Affiliations:** a School of Business and Economics, Maastricht University and UNU-MERIT/MGSoG, Maastricht, The Netherlands; b School of Business and Economics, Maastricht University and Maastricht School of Management, Maastricht, The Netherlands; c IZA-Institute for the Study of Labor, Bonn, Germany

**Keywords:** Entrepreneurship, Enterprise performance, Informal sector, Rural development, Self-employment, Small businesses, Sub-Saharan Africa

## Abstract

We report on the prevalence and patterns of non-farm enterprises in six sub-Saharan African countries, and study their performance in terms of labor productivity, survival and exit, using the World Bank’s Living Standards Measurement Study - Integrated Surveys on Agriculture (LSMS-ISA). Rural households operate enterprises due to both push and pull factors and tend to do so predominantly in easy-to-enter activities, such as sales and trade, rather than in activities that require higher starting costs, such as transport services, or educational investment, such as professional services. Labor productivity differs widely: rural and female-headed enterprises, those located further away from population centers, and businesses that operate intermittently have lower levels of labor productivity compared to urban and male-owned enterprises, or enterprises that operate throughout the year. Finally, rural enterprises exit the market primarily due to a lack of profitability or finance, and due to idiosyncratic shocks.

## 1 Introduction

A significant number of rural households in sub-Saharan Africa do not limit labor allocation to agriculture, but also operate and work in non-farm enterprises (Reardon et al., 2006).^1^ Over time the contribution of these enterprises to household incomes and employment has increased rather than decreased, as some development economists in the 1960s and 70s expected (Lanjouw and Lanjouw, 2001, Start, 2001, Haggblade et al., 2010). This contribution is unlikely to diminish in the future given that rural businesses will be needed to support the job creation for the roughly 170 million new job seekers entering Africa’s labor market between 2010 and 2020 (Fox and Pimhidzai, 2013). In this regard it is useful to have an up-to-date and accurate profile of the prevalence, patterns and performance of rural enterprises. So far most existing empirical work on African entrepreneurship is based on one-period, single-country and rather limited survey data. And although comprehensive research has been done to study the income diversification of rural households and the determinants thereof (see Davis et al., 2010, Davis et al., 2014), systematic knowledge on the performance of rural enterprises is virtually non-existent.

Hence, the contribution of this paper is twofold. First, it provides up-to-date and comparative evidence on the prevalence and patterns of rural enterprises. Second, it provides an empirical analysis of their performance, as well as a set of descriptive statistics on their survival and exit. We use the Living Standards Measurement Study - Integrated Surveys in Agriculture (LSMS-ISA) data set, a nationally representative data collection covering six countries over the period 2005 to 2013, namely Ethiopia, Malawi, Niger, Nigeria, Tanzania and Uganda. It is the first time, to the best of our knowledge, that this data set is used to study Africa’s rural enterprises.

Our main results are as follows. First, we confirm the prevalence of rural entrepreneurship as established in the existing literature. To be specific, we find that almost 42 percent of rural households operate an enterprise. Relevant determinants of this decision are household size, the experience of shocks, access to credit and markets, household wealth and various individual characteristics of the household head. Most households operate businesses in easier-to-enter activities, such as sales and trade, but fewer households in activities that require higher starting costs or educational investment. Second, rural and female-headed enterprises, those located further away from population centers, and businesses that operate intermittently, report lower levels of labor productivity than urban and male-owned enterprises, or enterprises that operate throughout the year. Education or the experience of a shock are further relevant factors affecting labor productivity. Third, we find that enterprises exit the market primarily due to a lack of profitability or finance, and due to idiosyncratic shocks.

The paper is structured as follows. In Section 2 we summarize the current state of knowledge on non-farm entrepreneurship in rural Africa. In Section 3 we describe the LSMS-ISA data set and its shortcomings, and present descriptive statistics on the prevalence and patterns of rural enterprises, followed by an analysis of the decision to operate a business in Section 4. In Section 5 we report our empirical findings on the performance of these enterprises, as well as descriptive statistics on survival and exit. The final section concludes.

## 2 Literature review

In the year 2000 Wiggens (2000) lamented that ‘little is known’ about Africa’s rural non-farm economy, beyond an ‘embryonic set of ideas’. Since then the embryonic set of ideas has been elaborated in more detail by scholars, particularly with respect to the decision of entering entrepreneurship, and the contribution of non-farm enterprises to household income and employment. The performance, survival and exit of these enterprises, however, have largely been neglected.

### 2.1 The decision to operate a business

The literature on the decision to enter entrepreneurship has identified both push (necessity) and pull (opportunity) factors (Herrington and Kelly, 2012). Pull factors include opportunities to earn an income during the lean season, while push factors include low incomes and negative shocks (Barrett et al., 2001). The households’ desire to maintain consumption in the face of risks and incomplete insurance and credit markets can motivate them to reduce their exposure to shocks by operating such an enterprise (Janvry and Sadoulet, 2006, Dercon, 2009).

This enterprise type, generally ‘family firms’, reflects the household’s exposure to risk. Family ties can provide informal insurance to households given limited social security and a high-risk environment (Bridges et al., 2013). For example, if a household member loses employment, this person’s labor supply is often absorbed into a family business (Bridges et al., 2013). While household members can be pushed into entrepreneurship, as growing families (i.e. surplus labor) put pressure on fixed farmland (Reardon, 1997, Reardon et al., 2006, Babatunde and Qaim, 2010), large households may also leverage more resources, such as labor and finance, that in turn facilitate entrepreneurship (Alsos et al., 2013).

Despite the household nature of enterprises, households in rural Africa do not always maximize a single, joint utility function (Ngenzebuke et al., 2014). Instead the decision-making takes place collectively (Chiappori, 1992), either in a cooperative or non-cooperative way (Manser and Brown, 1980). This means that full cooperation might be limited, and that biases against specific household members, for example women, can be expected (Serra, 2009). Female participation might not only be constrained by discrimination in financial and labor markets, but also due to ‘rigid social norms’ that influence their time-use (Minniti and Naudé, 2010). Nonetheless women have been found to be more likely to engage in the non-farm economy than men (Canagarajah et al., 2001, Rijkers and Costa, 2012, Ackah, 2013).

### 2.2 Contribution of household income

The vast majority of enterprises are small and informal businesses (Nagler and Naudé, 2014), with 95 percent of rural enterprises employing less than five workers (Haggblade et al., 1989). According to Davis et al. (2014) 44 percent of households in rural Africa participate in the non-farm economy, where self-employment contributes on average 15 percent to household income. ‘Farming remains the occupation of choice’ with at least 55 percent of household income deriving from agriculture (Davis et al., 2014 p. 26).

Another salient fact is that entrepreneurship in Africa contributes less to household income compared to other regions (Davis et al., 2010, Davis et al., 2014). For instance, Janvry and Sadoulet (2001) find that the non-farm economy contributes on average 55 percent to rural household income in Mexico, whereas Escobal (2001) reports a figure of 51 percent for Peru. Lanjouw and Lanjouw (2001) report 39 percent for Brazil, 41 percent for Chile, 50 percent for Colombia and 59 percent for Costa Rica. Shi et al. (2007) report 46 percent for China.

### 2.3 Performance

The literature on enterprise performance is largely focused on enterprises in developed economies (see e.g. Moretti, 2004, van Biesebroeck, 2005, Foster et al., 2008, Nichter and Goldmark, 2009, Bloom and van Reenen, 2010, Ali and Peerlings, 2011, Amin, 2011, Kinda et al., 2011, Martin et al., 2011, Saliola and Seker, 2011, Rijkers and Costa, 2012, Bloom et al., 2013). Only a few studies have analyzed enterprise performance in sub-Saharan Africa. These tend to focus either on formal or manufacturing enterprises and are overwhelmingly urban-based. For instance, Frazer, 2005, Bekele and Worku, 2008, Loening et al., 2008, Shiferaw, 2009, Klapper and Richmond, 2011 establish in their work that managerial and technical skills, finance and social networks, the macro-economic and business environment, as well as firm age and size are important determinants of firm performance in urban Africa. Empirical evidence on the performance of rural enterprises is however scarce.

In one of the few existing studies, Rijkers et al. (2010) analyze the productivity of manufacturing enterprises in Ethiopia, and find that rural enterprises are less productive than urban ones. They report an output per labor ratio for remote rural enterprises of 0.43, while it is 0.95 for enterprises in rural towns, and 2.30 for enterprises in urban areas (Rijkers et al., 2010 p.1282). Furthermore they point out that productivity levels are more dispersed in rural enterprises, and that female-headed enterprises are less productive than male-headed ones.

An important dimension of enterprise performance is survival. Although the general view is that rural enterprises face considerable constraints to grow and survive (Bekele and Worku, 2008), only a few empirical studies have analyzed this topic. For instance, McPherson (1995) finds that business size does not significantly affect enterprise survival in Botswana and Swaziland, but that larger firms are less likely to survive in Zimbabwe; findings that are in contrast with those found in developed countries. The author further finds that rural and female-owned enterprises are more likely to fail than urban or male-owned ones.

As far as location is concerned, Fafchamps and Shilpi (2003) find, using a non-African (Nepalese) sample, that the share of non-farm wage employment declines the further a household lives away from an urban center, suggesting that distance to a population center affects performance. There is a lack, however, of comparative research focusing on sub-Saharan Africa, with the exception of Owoo and Naudé (2016) who provide evidence on the spatial clustering of rural enterprise productivity in Ethiopia and Nigeria.

Summarizing the literature survey, we conclude that non-farm enterprises in rural Africa are small, informal businesses operated due to both necessity and opportunity, and contributing with a significant share to rural household incomes. However, they do not seem to be dynamic, nor to create the number of (non-family) jobs urgently needed, and furthermore tend to perform less well than urban-based, formal enterprises. In the remainder of this paper we explore whether this conclusion continues to be valid using the comparative data from the LSMS-ISA data set.

## 3 Data

The LSMS-ISA database results from nationally representative, cross-sectional and longitudinal surveys conducted by the World Bank in collaboration with national statistical offices in various countries in sub-Saharan Africa.^2^ The surveys cover six countries at the time of writing: Ethiopia, Malawi, Niger, Nigeria, Tanzania and Uganda. Cross-sectional data is currently available for all countries, and panel data for Nigeria, Tanzania and Uganda.

The surveys consist of three parts: a community questionnaire, an agricultural questionnaire and a household questionnaire. The community questionnaire collects community-level information including the communities’ access to public services and infrastructure, social networks, governance, and retail prices. The agricultural questionnaire collects information on crop production, storage and sales, land holdings, farming practices, input use and technology adaption, access to and use of services, infrastructure and natural resources, livestock, and fishery. Finally, the household questionnaire captures household demographics, migration, education, health and nutrition, food consumption and expenditure, non-food expenditure, employment, non-farm enterprises and further income sources, dwelling conditions, durable assets, and participation in projects and programs. The location of all households has been geo-referenced.

Despite the usefulness and strength of the data, which presents an improvement in the data collection and survey design for Africa, it is subject to a number of limitations. For instance, differences and inconsistencies in the content of questionnaires across countries only allow for limited comparability. Furthermore, measures of enterprise performance such as labor productivity, are partial and imprecise. It is also not possible to match the type of business activity with individual enterprise information, which limits the calculation of productivity dispersal across different types of businesses. A final limitation is that the surveys did not collect sufficient information on failed enterprises, preventing the use of survival analysis techniques.

## 4 Prevalence, patterns and determinants

### 4.1 Prevalence

Table 1 shows that almost 42 percent of the 24,551 rural households operate a non-farm enterprise (NFE). Overall, the sample comprises 11,064 enterprises in 8,115 rural households, resulting in an average of 1.36 enterprises per household. The country shares vary widely, from a relatively low share of 17 percent in Malawi to almost 62 percent in Niger.^3^

### 4.2 Patterns

The share of household income deriving from non-farm enterprises can be substantial^4^: 27 percent of households that are engaged in entrepreneurship derive 50 percent or more of their income from these activities, but only 5 percent of households all income, suggesting that most entrepreneurial activities are performed alongside other activities, most commonly agriculture.

**Fig. 1:**
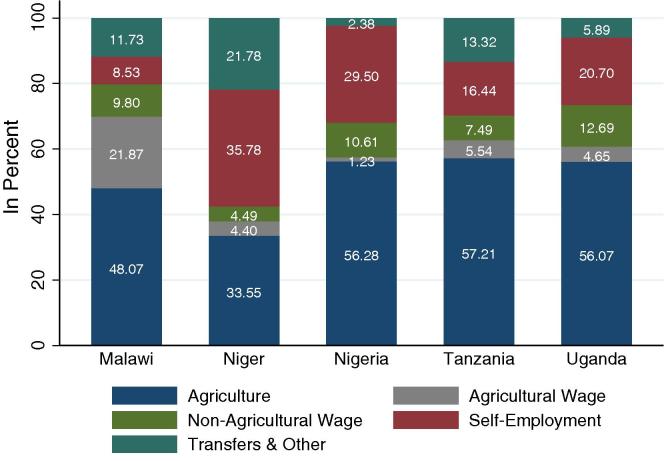
Contribution of activity to total household income. *Note(s)*: Weighted shares. Rural areas only.

We use the RIGA data set to calculate the shares of annual net household income by household activity for five countries in the sample.^5^ Fig. 1 indicates that the contribution of self-employment to household income varies widely. While it contributes less than 9 percent in Malawi, the share is approximately four times as high in Niger (almost 36 percent). Household income deriving from self-employment is smaller in rural than in urban areas (not included in Fig. 1). In urban areas self-employment contributes 22 percent to household income in Malawi, 48 percent in Niger, 53 percent in Nigeria, 43 percent in Tanzania and 33 percent in Uganda.

The vast majority of enterprises in the LSMS-ISA data set are small household enterprises in the informal economy. Over 80 percent do not employ any non-household worker, and less than 3 percent employ five or more workers.

Already two decades ago Bryceson (1996) pointed to the lack of data on occupational specialization in rural Africa. Given that this lack of data continues to persist, we provide an overview of the types of business activities that households operate, presenting data for three countries (where data availability permits) in Table 2.^6^ Many households operate businesses in easy-to-enter activities, such as sales and trade, compared to those venturing into activities that require higher starting costs, such as transport services, or that require educational investment, such as professional services. Agribusinesses constitute a noticeable share of rural business activities. Finally, many enterprises are classified under the category ‘other’, which are not further detailed in the surveys, pointing to the heterogeneity in the type of activities that rural households engage off-farm.

The differences between the countries in terms of business types are not large. In all three countries, trade and sales businesses take the largest share of business activities with around one third of all activities. Agribusiness is more prevalent in Ethiopia and Niger, non-agricultural activities take an important share in Ethiopia, while this business type takes a rather small share in Niger. In contrast, Niger has a higher share of businesses offering professional services.

### 4.3 Determinants

To identify the decision to operate a non-farm enterprise, we estimate a discrete choice model (probit model), and select our explanatory variables based on the occupational choice literature. Occupational choice models (see e.g. Lucas, 1978, Evans and Jovanovic, 1989, Murphy et al., 1991, Banerjee and Newman, 1993) have identified household and individual level determinants, which include the individual’s entrepreneurial skills, age and experience, the perceived relative rates of returns to self-employment, obstacles such as capital constraints, and factors that influence the opportunity costs of self-employment, including regulations and social protection.

Formally we estimate,

(1) $$\mathrm{Pr}(Y_{i}|v_{i}\text{,}w_{i}\text{,}x_{i}\text{,}z_{i})=\Phi (v_{i}^{\prime }\alpha +w_{i}^{\prime }\beta +x_{i}^{\prime }\gamma +z_{i}^{\prime }\delta )$$

where the dependent variable $Y_{i}$ is a binary variable equal to one if a household operates a non-farm enterprise, and zero if not. $v_{i}^{\prime }$ is a vector of individual characteristics including a constant, and comprises the variables gender, age, the marital status, and education (proxied by the ability to read & write) of the household head. $w_{i}^{\prime }$ is a vector of household characteristics including the number of adult household members, annual net household income, the number of rooms in the dwelling, and a binary variable if a household member has taken out credit over the past 12 months, indicating the possibility of accessing financial support. We also add the variable land size (in acres) per adult household member, where land can be either owned or rented. $x_{i}^{\prime }$ records whether or not the household has experienced food shortage or a shock over the last 12 months. Finally $z_{i}^{\prime }$ is a set of location variables (geo-variables), including the household’s distance to the next population center and annual precipitation.^7^ For the probit model we use a cross-sectional sample and take the last rounds available as of fall 2013.

We estimate the model for each country^8^ and present our results in Table 3, reporting average marginal effects (AME).^9^ The results show that various individual and household level characteristics have surprising outcomes. We find that the gender of the household head is not significant in any country, as well as land size per adult household member in most countries. We would have expected a negative correlation in both the case of gender (female) and land size. However, various variables are also in line with our expectations. Education, proxied by the ability to read & write, is generally positive and significant, and increases the likelihood to be entrepreneurial, as well as the number of adult household members indicating that surplus labor can be allocated to business operation. Variables that proxy wealth (income and number of rooms) generally increase the probability of operating an enterprise, suggesting that enterprises might be as well started due to opportunity.

One of the salient determinants of entrepreneurship in the literature is credit, or access to finance. Most studies concur that entrepreneurs often face liquidity constraints (Evans and Jovanovic, 1989, Hurst and Lusardi, 2004). Our results are consistent with the literature and show that access to credit is generally associated with a higher likelihood of operating an enterprise.

The results further indicate that households that experience food shortage over the past 12 months are more likely to operate an enterprise in Uganda, but are less likely to do so in Malawi and Niger. This finding suggests that the experience of food shortage can push households into entrepreneurship in times of necessity, but may also prevent them from entering the non-farm sector, depending on the country context and circumstances.

The shock variables are largely not significant, and have diverging associations with entrepreneurship when significant. The conclusion from this diversity of associations between risk and the likelihood of enterprise operation is that external risk factors have different and even opposing effects depending on country circumstances. These findings reflect that household risk-coping and risk-management strategies adjust to local circumstances.

Finally, we consider the local business environment and proximity to agglomerations, as proxies for access to markets (demand), as well as rainfall to account for agricultural conditions (Zezza et al., 2011). The regression results indicate that distance to the next population center has a negative and significant effect in Malawi and Uganda. Rainfall, a proxy for agricultural productivity, has contrasting associations where significant. Better rainfall, resulting in improved agricultural conditions, decreases the likelihood of enterprise operation in Nigeria, as more household members might be required to provide labor on farms. Interestingly, increased rainfall is associated with a higher likelihood of enterprise operation in Niger, maybe as a result of different farming practices. The results confirm that agglomeration and infrastructure, measuring potential market access and agricultural conditions, are significant determinants of rural enterprise operation.

## 5 Performance

We present our empirical evidence on the performance of rural non-farm enterprises in two subsections. First, we report our results on labor productivity, and second on seasonality, survival and exit.

### 5.1 Productivity

#### 5.1.1 Measurement

One of the most common measures of enterprise performance is its productivity. We calculate labor productivity, a partial productivity measure, as follows,

$$\text{labor productivity}=\frac{\text{average monthly sales}}{\text{number of workers}}$$

As average monthly sales is not available for all countries, we take total sales during the last month of operation in Malawi and Nigeria, and average gross revenues in Uganda. The number of workers include both household and non-household workers, and takes the value 1 if only the enterprise owner operates the business without further employees. Once labor productivity is calculated, we take the log of labor productivity for our estimates.

We are aware that this productivity measure is an approximation, and imprecise for a number of reasons. First, we do not take into account the enterprise output or profit due to a lack of cross-country data. Second, we do not have information about the time-use of workers, and do not know how many hours of work are allocated by each individual to the enterprise. This shortcoming is expected to be particularly relevant due to a large number of enterprises that operate less than 12 months per year, indicating seasonal operation which is probably connected to agricultural labor. And third, we assume that the self-reported variable itself is imprecise, as most enterprise are informal and do not always record sales or revenues, but provide rough estimates of the business volume. Due to data availability, this productivity measure is the best approximation that is currently at hand for this study to analyze labor productivity from a comparative point of view.

It is possible to calculate a more precise productivity estimate for Malawi, using enterprise profits instead of total sales in the numerator. To backup the results in this section, we generate kernel density estimates using profits, and compare them with the outcomes using sales. The results are shown in Appendix C, and largely confirm the findings of this section. However, the differences by location become smaller, and practically disappear when accounting for the gender of the enterprise owner. The appendix contains a more elaborate discussion.

In the case of Malawi the survey also includes questions on the time-use of workers. The enterprise section asks for the number of hours, days and months per year worked in the enterprise for household and non-household employees. However, the data is not precisely recorded, and only the number of months can be used to account for part-time labor supply, for example during the low season. We use this measure, in addition to the profitability of Malawian enterprises, and depict our results in Appendix C. Accounting for the time worked in the enterprise, the differences between urban and rural diminish further, but continue to be statistically significant at a 5 percent level, and at a 10 percent level when accounting for the gender of the enterprise owner.

#### 5.1.2 Dispersal

**Fig. 2:**
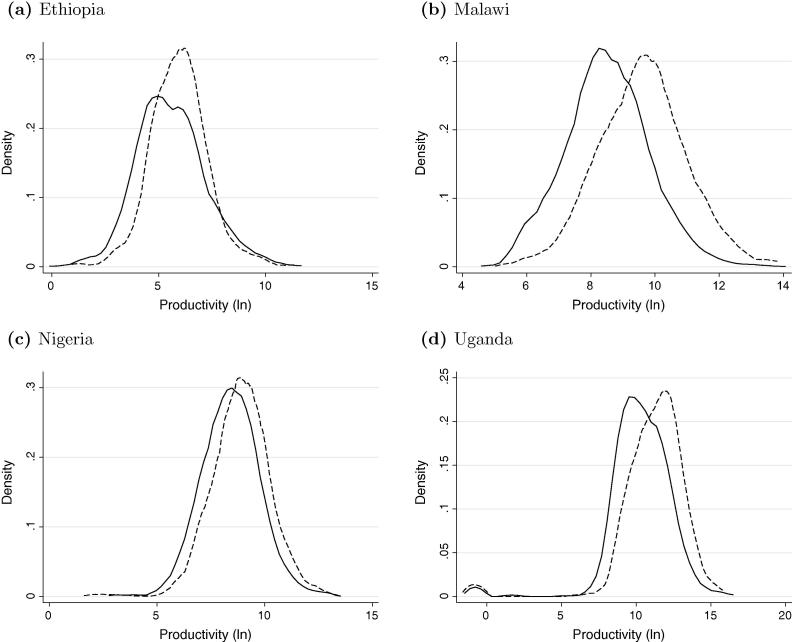
Productivity dispersal – by location. *Note(s)*: In (a)–(d) the continuous lines represent the productivity of enterprises that are located in rural areas and the dotted lines the productivity of enterprises that are located in urban areas.

The kernel density estimates of labor productivity by location are contained in Fig. 2. The figure shows that differences in labor productivity generally confirm our expectations. Urban enterprises are more productive than rural ones, with a more pronounced productivity gap in Malawi and Uganda.^10^

Although urban enterprises seem more productive on average, the simple dichotomy between rural and urban areas may be of limited use due to a large variation in Africa’s economic geography between deep rural, small towns and major urban areas. The potential importance of secondary towns and rural agglomerations has generally been underestimated. As Christiaensen et al. (2013) point out, productivity might be higher in metropolitan areas, but not all inhabitants from rural areas are or will be able to access these opportunities. They find that only one in seven people who escaped from poverty did so by migrating to a large city, but that one in two did so by migrating to a secondary town.

We are therefore interested in providing a finer analysis of how spatial location affects labor productivity. We use data from Uganda and analyze it with regard to the country’s division into four main regions and the capital city. These regions are distinct in terms of population density, business environment and history. Over the past ten years economic growth has been largely concentrated in the Central region that includes the capital city, while violent conflicts affected the Northern region between 1987 and 2006 (Blattmann et al., 2014). The Western region has also not escaped occasional (ethnic) conflicts (Espeland, 2007). This allows us to obtain some indication of how the local business environment may affect labor productivity, as regions that experienced a conflict are more likely to show a poorer business environment (Brueck et al., 2013).

**Fig. 3:**
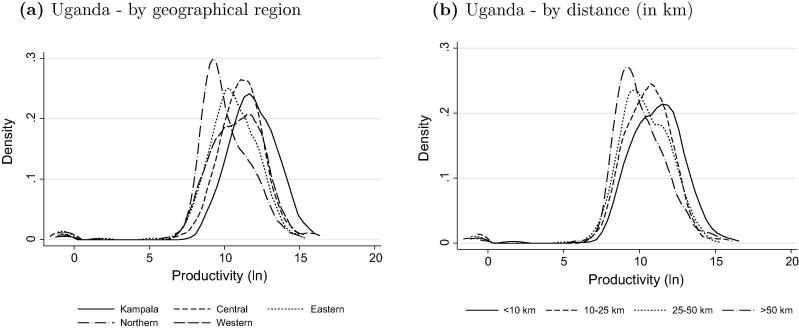
Productivity dispersal – by region and distance.

Fig. 3a confirms our expectations. Kampala reports the highest labor productivity, with the Central region following in terms of productivity level. The Northern region, with the lowest population density and a history of conflict, is home to the enterprises with the lowest productivity.

We also investigate whether distance to the closest population center has an effect on rural labor productivity. We again use data from Uganda and compare the density of labor productivity by various distance categories from the nearest population center. Fig. 3b shows that enterprises of households located up to 10 km from a population center are the most productive ones, followed by enterprises of households residing up to 25 km and 50 km away, respectively. If households are located more than 50 km away, the results show a significant productivity decline. These findings support the idea that rural secondary towns and cities, by providing links between rural areas and major cities, play an important role in the structural transformation and poverty reduction of agrarian societies.

**Fig. 7:**
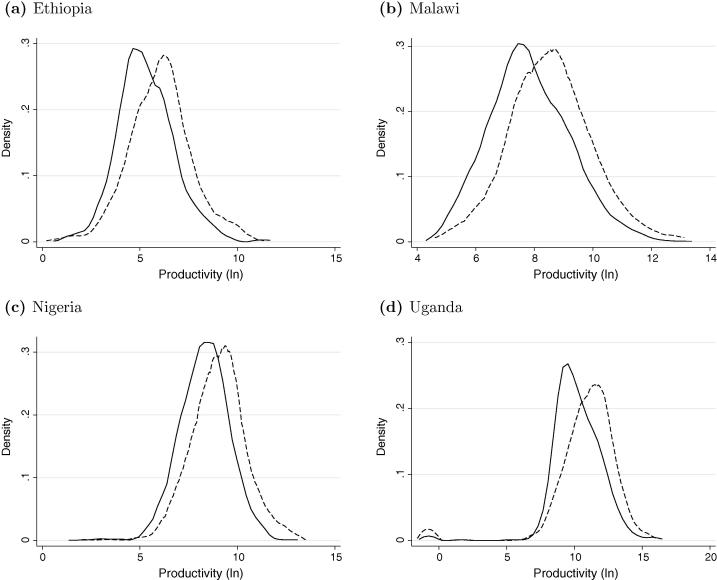
Productivity dispersal – by gender. *Note(s)*: In (a)–(d) the continuous lines represent the productivity of enterprises with a female enterprise owner and the dotted lines the productivity of enterprises with a male enterprise owner. Authors’ own calculations based on LSMS-ISA data.

**Fig. 8:**
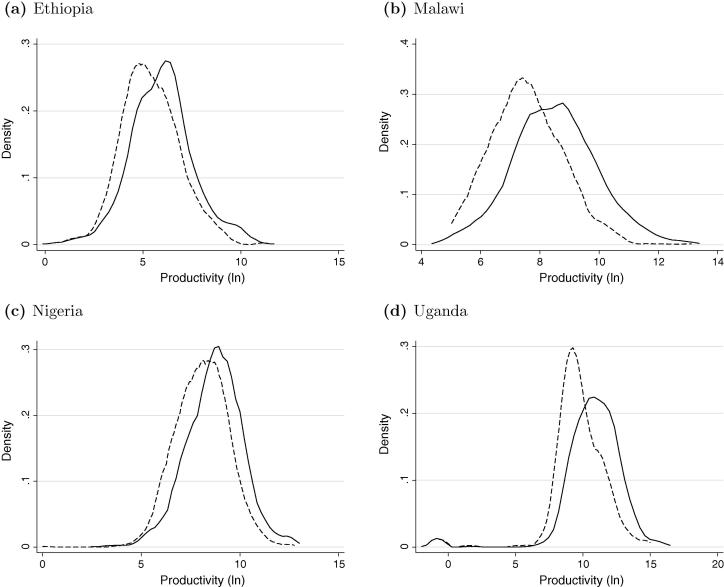
Productivity dispersal – by ability to read & write. *Note(s)*: In (a)–(d) the continuous lines represent the productivity of enterprises where the enterprise owner has the ability to read & write and the dotted lines the productivity of enterprises with an illiterate enterprise owner. Authors’ own calculations based on LSMS-ISA data.

**Fig. 9:**
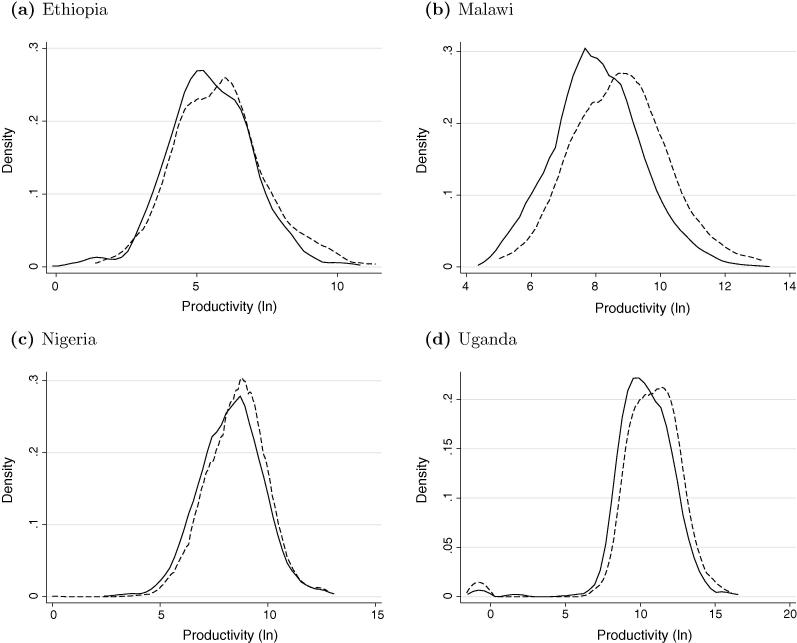
Productivity dispersal – by shock experience. *Note(s)*: In (a)–(d) the continuous lines represent the productivity of enterprises where the corresponding household experienced a shock during the 12 previous months and the dotted lines the productivity of enterprises where the corresponding household did not experience a shock. Authors’ own calculations based on LSMS-ISA data.

We further depict the productivity dispersal by gender, education (proxied by the ability to read and write) and the experience of shocks in Fig. 7, Fig. 8, Fig. 9.^11^ Enterprises with a male owner are on average more productive than enterprises with a female owner in all four countries. A possible explanation could lie in the fact that businesses are time-consuming to operate, and women tend to be more time-constrained due to household duties (see also Palacios-López and López, 2014). The productivity dispersal by the ability to read and write shows that literate enterprise owners operate more productive enterprises. However, the ability to read and write is an imprecise approximation to the educational level of the entrepreneur, capturing individuals with primary education, but also individuals with more comprehensive education, for example secondary schooling. However, given that we expect managerial capacity to be important for enterprise productivity (see e.g. Sonobe et al., 2012), the results provide some support in this regard.

Finally, the productivity dispersal by the experience of a shock during the last 12 months preceding the survey is reported. As expected, the experience of shocks largely shows a negative association with labor productivity. While the difference is minor in Ethiopia and Nigeria, it is more pronounced in Malawi and Uganda.

#### 5.1.3 Determinants

To estimate the determinants of enterprise performance, proxied by our measure of labor productivity, we account for possible sample selection effects using a Heckmann selection model. Given that we can only observe the labor productivity of enterprises that are actually operating, we might get biased estimates if we do not control for the selection effect in the decision to enter entrepreneurship.

Formally we estimate,

(2) $$z_{i}^{*}=w_{i}\gamma +u_{i}$$

representing the selection stage of the model, where $z_{i}^{*}$ determines whether or not an enterprise is operated, thus $z_{i}=1$ if $z_{i}^{*}>0$ and $z_{i}=0$ if $z_{i}^{*}⩽0$. $w_{i}$ is a vector containing the possible determinants of enterprise operation.

Once $z_{i}$ is known, the outcome stage with the dependent variable “log of labor productivity” can be modeled as,

(3) $$y_{i}^{*}=x_{i}\beta +\varepsilon_{i}$$

with $y_{i}=y_{i}^{*}$ if $z_{i}=1$ and $y_{i}$ not observed if $z_{i}=0$.

$x_{i}$ is a vector containing the possible determinants of labor productivity.

In the selection stage we take the individual characteristics of the household head, and include the variables gender, age and education. As household characteristics, we use access to credit, experience of shocks, and land size (in acres) per adult household member, as well as the location characteristics distance to the next population center, rural and agro-ecological zone. As the selection variable we use number of adult household members, since larger households have surplus labor available to allocate to entrepreneurial activities. We make the assumption that the number of adult household members do not influence productivity.^12^

In the outcome stage we take the individual characteristics of the enterprise owner (instead of the household head), and include otherwise the same variables as in the selection stage. Additionally, we take information of the enterprise, such a months in operation and the information if credit was used to expand the business.

For the Heckman selection model we use a cross-sectional sample and take the last rounds available as of fall 2013. Table 4 reports the regression results, including the selection and outcome stage.

The first stage, the selection into entrepreneurship, is reported in the lower part of the table. For the selection stage we make the assumption that the number of adults in a household selects households into this sector, as surplus labor is allocated into the enterprises. The results of the second stage, the outcome stage, reported in the upper part of the table, can then be interpreted as though we observed data for all households in the sample.

The number of adults, the selection variable, is significant and positive in all four countries. The significance test also shows that the selection stage is significant for all countries included, suggesting that we use an appropriate estimation method.

We find a lower probability of entering the sector for households located in rural areas. Most other variable results are in line with the outcomes shown in 3, with the exception of land size that is significant in three countries with opposing associations. While more land leads to higher levels of entrepreneurship in Malawi and Uganda, farming activities seem to reduce the likelihood of enterprise operation in Ethiopia.

Results in the outcome stage show that female-owned enterprises are less productive than male-owned enterprises in most countries, while the effect of age is not significant. We further find that the effect of education on labor productivity is positive and significant, and that access to credit is not or only marginally significant, a surprising fact. The effect of being located in a rural area is negative in Malawi and Nigeria. Shocks (reflecting risk) have a negative impact in most cases. While distance from a population center lowers the probability of households entering entrepreneurship, it is associated with higher labor productivity in Malawi, but with lower productivity in Nigeria and Uganda. A possible explanation could be border effects in the case of Malawi.

### 5.2 Seasonality, survival and exit

Our interest in enterprise performance is also based on the assumption that more productive enterprises are more likely to operate continuously over the year, to grow and survive, and to create the quantity and quality of employment necessary for development and structural change in rural Africa. In this section we analyze, to the extent possible given data limitations, the seasonal operation patterns, as well as survival and exit of these enterprises. The LSMS-ISA data is not ideal to study this area of enterprise behavior as the surveys were not designed as enterprise surveys. Given the subsequent lack of information on failed enterprises, we present a set of descriptive statistics to obtain a provisional overview on seasonality, survival and exit in the rural non-farm enterprise sector.

**Fig. 4:**
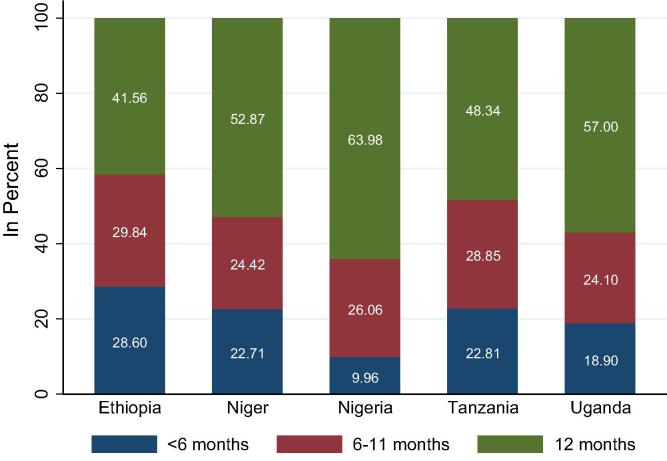
Months in operation. *Note(s)*: Malawi not reported due to a lack of data. Enterprises that are less than one year in operation are excluded. Rural areas only.

To start the analysis, we account for possible seasonality patterns in the operation of enterprises. The LSMS-ISA data captures the number of months per year a rural enterprise was operating in the year preceding the survey. Fig. 4 shows that a significant proportion was operating for less than 6 or 12 months per year. Between 42 and 64 percent of all enterprises operate continuously during the whole year, with the highest percentage found in Nigeria.

From the survey data we also calculate these shares for enterprises operating in urban areas. Table 5 shows that proportionately more urban enterprises operate continuously over the year compared to rural ones, suggesting that seasonality has a significant impact on the dynamics of rural entrepreneurship. If enterprises that are operated intermittently absorb idle work, they can be expected to remain small and informal, with a low productivity and without creating additional jobs.

**Fig. 5:**
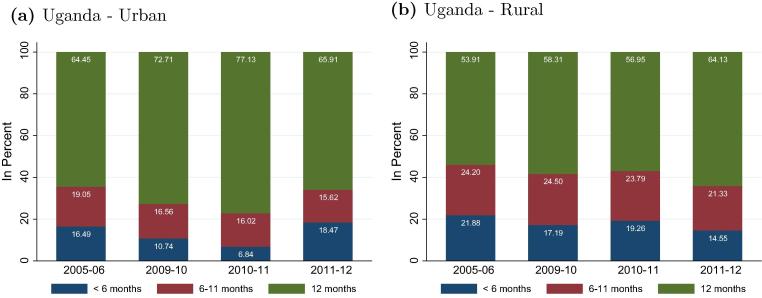
Months in operation – by location. *Note(s)*: Survey weights included.

Fig. 5 depicts the months of enterprise operation over time using data from Uganda. We notice the difference between rural and urban enterprises. In all four survey years the share of enterprises operating throughout the year is lower in rural than in urban areas.

**Fig. 6:**
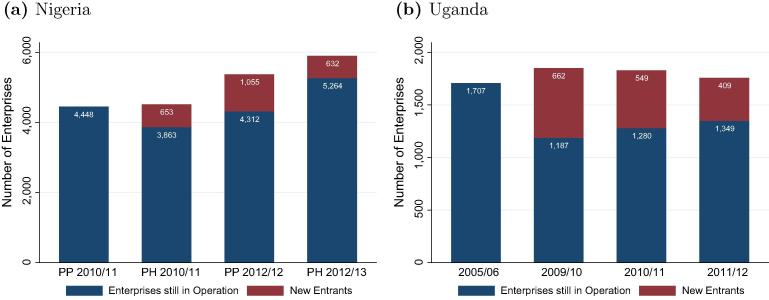
Entries and exits of non-farm enterprises. *Note(s)*: PP = Post-Planting, PH = Post-Harvest. Rural areas only.

The surveys also capture information about the numbers of enterprises operated across the survey waves. Fig. 6 shows the number of enterprises counted for Nigeria and Uganda in each of the survey years. In Nigeria the number of new business activities are accounted for in one specific question, in Uganda enterprises from previous rounds that are no longer operating are included in the consecutive rounds as ‘empty’ observations. This data provides insights of entry and exit numbers into and from the non-farm enterprise sector. In the second, third and fourth survey rounds we include the number of enterprises that ‘survived’ in between the surveys, with the previous survey round as the baseline survey.

The number of enterprises has continuously increased in Nigeria, while it has been more stable in Uganda. We note that numbers of the same or close survey years are more similar, as less time has passed for entry and exit. Nigeria has not only experienced a steady increase, but also has a lower share of new entrants compared to Uganda, suggesting that entrepreneurship is a more stable activity. The country has also the highest share of enterprises that operate continuously throughout the year in rural areas.

Finally we investigate why enterprises exit the market. In Uganda the households were questioned why their enterprise stopped operations during the previous year. We summarize their responses in Table 6, distinguishing between rural and urban enterprises. The most important reasons of market exit are low profitability, lack of finance, as well as the impact of idiosyncratic shocks such as death or illness in the family, and unreliably supplies. Labor related reasons (idiosyncratic shocks) are more pronounced in rural areas, and a lack of finance plays a more important role in urban areas. Idiosyncratic shocks could be more pronounced in rural areas due to the nature of enterprises, which have less often workers employed in the business activities beyond the enterprise owner.^13^

Among the enterprises that were discontinued during the previous twelve months in Uganda, respondents were further asked whether they were planning to restart their enterprise. In 2010/11 57.34 percent did not plan a restart, 35.66 percent considered it probable, and the remaining 6.99 percent were certain of retaking operation. In 2011/12 73.19 percent did not plan a restart, 24.54 percent considered it probable, and the remaining 2.27 percent were certain of retaking operation.^14^ Generally enterprises were discontinued for good, indicating that many enterprises in rural Uganda are created, operated for a while and stopped. However, a share of firms also indicated plans to restart operations. This suggests the influence of seasonality, as well as business operation as a secondary activity that functions as some form of insurance in times of economic necessity. Furthermore unreliable financing might impede the continuation of enterprises, forcing the enterprise owner to wait for new income sources to continue operations.

## 6 Summary and concluding remarks

In this paper we provide a comparative overview of non-farm entrepreneurship in rural sub-Saharan Africa using the recent LSMS-ISA survey data. In particular, we focus on (i) the patterns and determinants of rural enterprise operation, (ii) their performance, and (iii) their survival and exit. In the remainder of this section we first summarize the results, before suggesting some policy implications.

First, we find evidence that households operate enterprises due to both push and pull factors. On the one hand, the necessity to cope and manage risks can push households into entrepreneurship. Given a lack of social protection and insurance schemes, rural households need to manage shocks, deal with surplus household labor, and respond to seasonality. Operating an enterprise can present a strategy to cope with these kinds of uncertainties. The necessity motivation is reflected in the nature of rural enterprises as small, informal household businesses that are often operated for only a portion of the year, and in easy-to-enter sectors or activities. On the other hand, some households do seize opportunities in the non-farm economy, as illustrated by a positive association of variables capturing higher incomes and wealth, and enterprise operation. In addition, our findings suggest that a shorter distance to markets and a favorable local infrastructure facilitate enterprise operation, indicating that business opportunities exist in rural areas.

Second, our results suggest a link between a household’s motivation to operate an enterprise and its subsequent performance. Enterprises operated by necessity, e.g. due to shocks, are on average less productive than enterprises operated as a result of the household utilizing an opportunity. We further find that rural enterprises are on average less productive than their urban counterparts, an expected outcome. Moreover, enterprises located in regions that have been subject to a history of violent conflict show a lower productivity level. With regard to individual level determinants of labor productivity, we find that female-owned enterprises are less productive than male-owned enterprises, although we may underestimate their performance due to the constrained time-use of women.

Third, we find that enterprises do not always operate throughout the year, and that many exit the market. In rural Africa a large number of enterprises are occasional enterprises that function for only a portion of the year. They are more often operating intermittently compared to their urban counterparts, which can be explained by seasonal labor requirements in agriculture. The most important reasons that households reported for enterprise exit in Uganda are economic factors, such as a lack of profitability and a lack of finance. We also find that more enterprises in rural areas cease operations due to idiosyncratic shocks compared to their urban counterparts. This reflects the risky environment in which they operate, and the small size of their businesses. However, enterprises are not always shut down for good, but a significant share of former enterprise owners consider restarting business activities in the near future.

Our results provide some justification for policies that support enterprise owners, such as access to credit to expand business activities and the development of local infrastructure. However, such policies are already part of most entrepreneurship development programs in sub-Saharan Africa, making it a trivial recommendation. Less trivial and also more difficult to implement is the suggestion to provide more concentrated support for enterprises with high growth potential due to a large heterogeneity in enterprise performance. Hence it is crucial to identify and support highly-talented (and younger) entrepreneurs that have the potential to take on more risky, but also more productive types of businesses, and who will locate their activities where positive spillovers can best be generated. What may be so far missing or inadequate in terms of enterprise policies are measures that can cushion shocks and protect households from negative external events, for example (micro)-insurance or social protection schemes. Such policies can help households to avoid operating unsustainable types of businesses, such as selling seeds or livestock, or prevent well-functioning enterprises from exiting operations. We expect that policies that tackle the difficulties of rural entrepreneurship from both angles, i.e. support entrepreneurs and cushion households from negative external events, will have a more pronounced impact on rural enterprise creation, productivity, and survival.

## Figures and Tables

**Table 7:** Prevalence of non-farm enterprises in urban Africa.

Country	Nr of HH surveyed	HH with NFE	in % weighted	Nr of NFEs	Avg Nr of NFE/HH
Ethiopia	503	293	56.11	370	1.26
Malawi	2233	817	35.11	938	1.15
Niger	1538	998	67.78	1539	1.54
Nigeria	1620	1,108	70.50	1760	1.59
Tanzania	1295	746	60.28	960	1.29
Uganda	611	354	54.21	572	1.62

Total	7297	4023	64.59	11,064	1.36

*Note(s)*: Weighted country shares are calculated using survey weights, the total share includes population weight.

**Table 8:** Summary statistics – probit model.

Household	Ethiopia		Malawi		Niger		Nigeria		Tanzania		Uganda	
operates a NFE	No	Yes	No	Yes	No	Yes	No	Yes	No	Yes	No	Yes
Female	0.20	0.18	**0.26**	**0.19**	0.11	0.09	**0.17**	**0.12**	0.25	0.24	**0.31**	**0.25**
Age	**45.37**	**41.77**	**43.50**	**39.84**	44.19	44.96	**51.38**	**48.98**	**48.95**	**44.99**	**46.28**	**42.33**
Married	0.81	0.82	**0.72**	**0.81**	0.90	0.91	**0.76**	**0.85**	0.57	0.54	**0.68**	**0.79**
Read & write	**0.40**	**0.49**	**0.58**	**0.71**	0.25	0.31	**0.48**	**0.63**	**0.64**	**0.74**	0.63	0.64
Number of adults	2.71	2.67	**2.37**	**2.53**	2.90	3.09	**2.91**	**3.34**	**2.83**	**3.12**	**3.26**	**3.64**
Land size	**1.24**	**0.90**	0.82	0.84	5.24	5.47	0.73	0.61	1.77	1.69	0.82	1.06
Income					**0.50**	**0.89**	**0.93**	**0.64**	**0.56**	**0.87**	**0.50**	**0.82**
Rooms	**1.64**	**1.81**	**2.44**	**2.63**	2.50	2.76			**3.37**	**3.82**	2.80	2.84
Credit	**0.26**	**0.37**	**0.11**	**0.19**					0.08	0.11		
Food shortage	0.32	0.37	**0.52**	**0.47**	**0.47**	**0.38**	0.26	0.27	0.21	0.21	0.22	0.27
Shock (idiosyn.)	0.18	0.20	**0.27**	**0.38**	0.22	0.21	0.23	0.20	0.17	0.19	**0.15**	**0.19**
Shock (price)	0.33	0.37	**0.41**	**0.46**	0.29	0.34	0.22	0.24	0.48	0.44	0.02	0.01
Shock (geogr.)	0.24	0.22	0.48	0.52	0.45	0.40	0.14	0.11	0.18	0.17	**0.29**	**0.35**
Shock (other)	0.02	0.04	0.02	0.02	**0.15**	**0.21**	0.01	0.01	0.01	0.00	0.02	0.02
Distance	0.37	0.33	**0.38**	**0.35**	0.66	0.62	0.24	0.23	0.54	0.53	0.25	0.25
Precipitation	1.17	1.21	**1.07**	**1.05**	**0.38**	**0.40**	1.47	1.42	1.07	1.07	1.24	1.22

*N*	2547	919	8283	1755	1003	1427	1673	1707	1761	1241	1152	953

*Notes*: Survey weights included. Coefficients in bold indicate differences between households with and without an enterprise that are significant at a 5 percent level. Rural areas only.

**Table 9:** Summary statistics – Heckman selection model (Ethiopia and Malawi).

	Ethiopia			Malawi		
	Obs	Mean	St dev	Obs	Mean	St dev
**Individual characteristics**						
*Household head*						
Female	4150	0.25	0.43	12,506	0.24	0.43
Age	4146	43.96	15.64	12,506	42.08	16.07
Read & write	4131	0.41	0.49	12,506	0.68	0.47

*Enterprise owner*						
Female	1344	0.51	0.50	2802	0.38	0.48
Age	1342	35.76	12.60	2802	37.06	12.22
Read & write	1336	0.48	0.50	2802	0.77	0.42

**Household characteristics**						
Number of adults	4159	4.13	2.16	12,506	2.47	1.22
Rural	4159	0.86	0.34	12,506	0.81	0.39
Credit	4087	0.25	0.42	12,506	0.13	0.34
Shock	4159	0.47	0.50	12,505	0.69	0.46
Land size	4149	0.86	1.73	12,506	0.71	0.79
Distance	4159	0.41	0.34	12,506	0.34	0.23

**Enterprise characteristics**						
Firm size	1470	2.25	1.86	2809	2.59	1.73
Months in operation	1332	7.85	3.98			

**Table 10:** Summary statistics – Heckman selection model (Nigeria and Uganda).

	Nigeria			Uganda		
	Obs	Mean	St Dev	Obs	Mean	St Dev
**Individual characteristics**						
*Household head*						
Female	6555	0.13	0.33	3508	0.29	0.46
Age	6537	49.36	14.81	3506	45.68	14.76
Read & write	6543	0.64	0.48	3395	0.66	0.47

*Enterprise owner*						
Female	4364	0.57	0.50	1909	0.52	0.50
Age	4358	40.86	13.84	1907	39.90	12.50
Read & write	4354	0.63	0.48	1895	0.74	0.44

**Household characteristics**						
Number of adults	6555	3.35	1.89	3508	4.10	2.51
Rural	6558	0.66	0.47	3511	0.76	0.43
Shock	6558	0.31	0.46	3444	0.46	0.50
Land size	6555	0.58	2.43	3508	0.84	2.41
Distance	6558	0.20	0.20	3102	0.22	0.19

**Enterprise characteristics**						
Firm size	4318	2.24	1.57	1525	3.14	1.89
Months in operation	4323	10.46	2.74	1529	9.48	3.57

**Table:** 

Definition of variables	
Female	1 if female
Age	In years
Married	1 if married (monogamous or polygamous)
Read & write	1 if individual can read and write in any language
Number of adults	Number of adults in the household age 15 and older
Land size	Land size in acres per adult households member
Income	Annual net household income in 1000’s of USD
Rooms	Number of rooms in the household
Credit	1 if household has access to credit. This variable is defined as general access to credit, and does not further specify for which purpose the household used it
Food shortage	1 if household experienced food shortage (self-reported variable)
Shock	1 if household experienced a shock. Shocks can be idiosyncratic (e.g. death or illness of a household member), related to prices (e.g. increase in the price level of certain goods and services), related to agriculture (e.g. droughts or floods), or other types of shocks (not further specified in the questionnaire)
Distance	Defined as distance to next population center of 20,000 or more inhabitants, in 100’s of km
Precipitation	Annual precipitation in 1000’s of mm
Months in operation	Number of months per year (12 months) an enterprise was in operation
Agro-ecological zones	Tropic-warm or tropic-cool, combined with different precipitation levels: arid, semi arid, sub humid, humid
Firm size	Number of workers in the enterprise

**Fig. 10:**
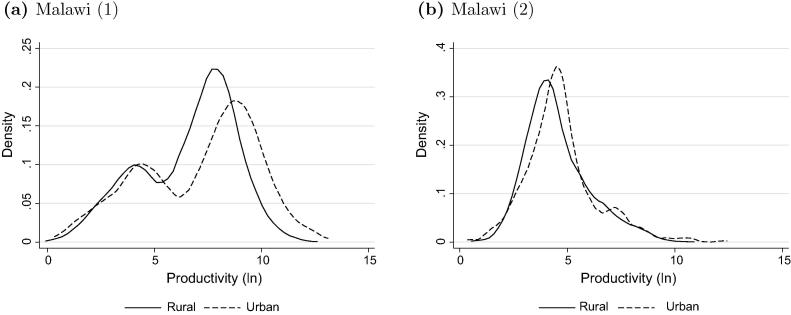
Productivity dispersal – by location. *Note(s)*: In (a) and (b) the continuous lines represent the productivity of rural enterprises and the dotted lines the productivity of urban enterprises. Malawi (1) shows the productivity measure using profit in the numerator, and Malawi (2) additionally makes use of time-use information of workers in the denominator.

**Fig. 11:**
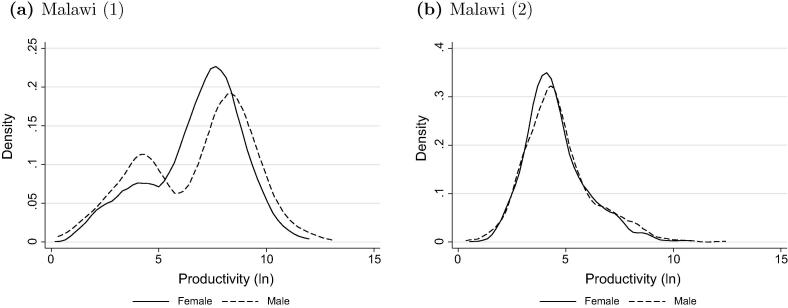
Productivity dispersal – by gender. *Note(s)*: In (a) and (b) the continuous lines represent the productivity of enterprises with a female enterprise owner and the dotted lines the productivity of enterprises with a male enterprise owner. Malawi (1) shows the productivity measure using profit in the numerator, and Malawi (2) additionally makes use of time-use information of workers in the denominator.

**Table 1:** Prevalence of rural non-farm enterprises.

Country	Nr of HH surveyed	HH with NFE	in % weighted	Nr of NFEs	Avg Nr of NFE/HH
Ethiopia	3466	919	22.87	1112	1.21
Malawi	10,038	1755	16.88	1872	1.07
Niger	2430	1427	61.73	2188	1.53
Nigeria	3380	1707	52.62	2688	1.57
Tanzania	2629	1061	38.65	1363	1.26
Uganda	2105	953	42.24	1471	1.54

Total	24,551	8115	41.63	11,064	1.36

*Note(s)*: Weighted country shares are calculated using survey weights, the total share includes the population weight. Rural areas only.

**Table 2:** Type of business activity.

Activity	Ethiopia	Malawi	Niger
Trade and sales	31.49	35.64	35.42
Agricultural business	26.31	20.09	26.62
Non-agricultural business	25.69	18.06	7.40
Professional services	1.12	0.53	4.28
Bar or restaurant	0.48	2.40	1.11
Transport	1.23	2.56	1.37
Other	13.69	20.72	23.81

Total	100	100	100

*Note(s)*: Weighted shares. Rural areas only.

**Table 3:** Probit regressions – by country.

Dependent	(1)	(2)	(3)	(4)	(5)	(6)
NFE	Ethiopia	Malawi	Niger	Nigeria	Tanzania	Uganda
Female	−0.031	0.003	−0.049	0.045	0.036	−0.016
	(0.04)	(0.02)	(0.06)	(0.10)	(0.03)	(0.04)
Age	−0.002∗∗	−0.001∗∗	0.001	−0.003∗∗	−0.004∗∗∗	−0.004∗∗∗
	(0.00)	(0.00)	(0.00)	(0.00)	(0.00)	(0.00)
Married	−0.032	0.037∗∗	−0.041	0.084	−0.061∗∗	0.074∗
	(0.04)	(0.02)	(0.06)	(0.08)	(0.03)	(0.04)
Read & write	0.031	0.040∗∗∗	0.030	0.126∗∗∗	0.054∗	−0.022
	(0.02)	(0.01)	(0.04)	(0.04)	(0.03)	(0.03)
Number of adults	−0.015	0.011∗	−0.020	0.054∗∗∗	0.032∗∗∗	0.035∗∗∗
	(0.01)	(0.01)	(0.01)	(0.01)	(0.01)	(0.01)
Land size	−0.032∗∗∗	0.009	0.001	−0.005	−0.001	0.011∗∗
	(0.01)	(0.01)	(0.00)	(0.01)	(0.00)	(0.01)
Income in USD			0.152∗∗∗	−0.017∗∗	0.039∗∗∗	0.092∗∗∗
			(0.02)	(0.01)	(0.01)	(0.02)
Rooms	0.036∗∗∗	0.008∗∗	0.007		0.023∗∗∗	−0.005
	(0.01)	(0.00)	(0.01)		(0.01)	(0.01)
Credit	0.089∗∗∗	0.071∗∗∗			0.021	
	(0.02)	(0.01)			(0.04)	
Food shortage	0.033	−0.028∗∗∗	−0.075∗∗∗	0.032	−0.006	0.091∗∗
	(0.03)	(0.01)	(0.02)	(0.04)	(0.03)	(0.04)
Shock (idiosyn.)	0.033	0.062∗∗∗	0.003	−0.036	0.048	0.060∗
	(0.02)	(0.01)	(0.04)	(0.04)	(0.03)	(0.03)
Shock (price)	0.009	0.001	0.024	0.022	−0.044	−0.167∗∗
	(0.03)	(0.01)	(0.03)	(0.04)	(0.03)	(0.07)
Shock (geogr.)	−0.023	0.021∗∗	−0.015	−0.069	0.003	0.051
	(0.03)	(0.01)	(0.03)	(0.04)	(0.03)	(0.03)
Shock (other)	0.135∗	0.021	0.097∗∗∗	0.028	−0.019	−0.035
	(0.08)	(0.04)	(0.03)	(0.10)	(0.15)	(0.09)
Distance	−0.004	−0.110∗∗∗	−0.080	−0.154	−0.019	−0.242∗∗
	(0.07)	(0.03)	(0.07)	(0.14)	(0.04)	(0.12)
Precipitation	0.033	−0.030	0.619∗∗	−0.065∗	−0.013	0.067
	(0.04)	(0.02)	(0.29)	(0.03)	(0.04)	(0.09)

*N*	3367	10,017	2430	1074	2579	1958

*Note(s)*: Standard errors in parentheses. Average marginal effects are reported. Rural areas only.

∗ p<0.1.

∗∗ p<0.05.

∗∗∗ p<0.01.

**Table 4:** Heckman selection model.

	(1)	(2)	(3)	(4)
	Ethiopia	Malawi	Nigeria	Uganda
*(ln) Productivity*				
Rural	0.213	−0.602∗∗∗	−0.179∗∗	0.402
	(0.24)	(0.12)	(0.07)	(0.30)
Female	−0.661∗∗∗	−0.575∗∗∗	−0.313∗∗∗	−0.220
	(0.14)	(0.07)	(0.05)	(0.15)
Age	0.004	0.000	−0.003	0.003
	(0.01)	(0.00)	(0.00)	(0.01)
Read & write	0.353∗∗	0.338∗∗∗	0.216∗∗∗	0.628∗∗∗
	(0.16)	(0.08)	(0.07)	(0.17)
Credit	0.047	−0.194∗		
	(0.17)	(0.10)		
Shock	−0.327∗∗	−0.418∗∗∗	−0.129∗	−0.252
	(0.15)	(0.08)	(0.07)	(0.19)
Land size	−0.092	0.015	−0.006∗	−0.024∗
	(0.08)	(0.05)	(0.00)	(0.01)
Months in operation	0.043∗∗		0.063∗∗∗	0.090∗∗∗
	(0.02)		(0.01)	(0.02)
Distance	−0.164	0.524∗∗∗	−0.307∗	−1.529∗∗∗
	(0.29)	(0.17)	(0.17)	(0.57)

*NFE*				
Number of adults	0.046∗∗	0.120∗∗∗	0.143∗∗∗	0.072∗∗∗
	(0.02)	(0.01)	(0.02)	(0.01)
Rural	−0.912∗∗∗	−0.469∗∗∗	−0.460∗∗∗	−0.368∗∗∗
	(0.09)	(0.06)	(0.05)	(0.09)
Female	0.094	−0.053	−0.154∗∗	−0.051
	(0.09)	(0.04)	(0.07)	(0.05)
Age	−0.012∗∗∗	−0.008∗∗∗	−0.006∗∗∗	−0.013∗∗∗
	(0.00)	(0.00)	(0.00)	(0.00)
Read & write	0.169∗∗	0.243∗∗∗	0.180∗∗∗	0.004
	(0.08)	(0.04)	(0.05)	(0.05)
Credit	0.340∗∗∗	0.254∗∗∗		
	(0.08)	(0.05)		
Shock	0.048	0.079∗∗	−0.023	0.097
	(0.07)	(0.04)	(0.05)	(0.07)
Land size	−0.123∗∗∗	0.055∗∗∗	0.000	0.024∗∗∗
	(0.04)	(0.02)	(0.01)	(0.01)
Distance	−0.266∗∗	−0.328∗∗∗	−0.118	0.203
	(0.13)	(0.09)	(0.13)	(0.20)

Agro-ecological zone	Yes	Yes	Yes	Yes
*N*	3889	12,496	5840	2698
rho	−0.284	−0.615	−0.474	−0.930
sigma	1.568	1.506	1.367	2.950
lambda	−0.445	−0.926	−0.649	−2.744
Prob > chi2	0.101	0.000	0.000	0.000

*Note(s)*: Standard errors in parentheses. Household weights included. Clustered at the household level.

∗ p<0.1.

∗∗ p<0.05.

∗∗∗ p<0.01.

**Table 5:** Months in operation – by location.

	Niger		Nigeria		Tanzania		Uganda	
	Rural	Urban	Rural	Urban	Rural	Urban	Rural	Urban
<6	23	9	10	7	23	12	19	7
6–11	24	16	26	24	29	25	24	16
12	53	75	64	69	48	63	57	77

*Note(s)*: Ethiopia excluded, since urban areas were not surveyed. Survey weights included.

**Table 6:** Reasons for enterprise exit.

Reason	2010–2011		2011–2012	
	Urban	Rural	Urban	Rural
Insecurity or theft	2.95	4.10	3.28	0.19
Lack of supply (inputs or raw material)	9.00	7.52	4.74	7.43
Lack of demand	5.14	6.04	5.84	1.50
Economic factors (profitability)	27.59	32.93	19.09	15.72
Technical issues	0.46	0.62	0.89	0.76
Labor related (death or illness)	5.57	9.00	5.68	7.07
Government regulation				0.89
Competition	1.79	1.67		3.30
Lack of electricity		0.15		
Lack of space or premises	0.55	0.29	0.43	1.47
Lack of transport	2.97	0.81		1.11
Lack of finance	29.33	23.59	34.63	31.37
Other	14.65	13.30	25.41	29.16

Number of observations	97	314	84	273

*Note(s)*: Survey weights included.

## Appendix C Additional productivity calculations

These figures present the kernel density estimates of an alternate and more precise measure of productivity that is possible to calculate for Malawi. In Fig. 10a we use enterprise profit in the numerator and number of workers in the denominator. For Malawi it is also possible to provide a finer-tuned productivity measure, since the questionnaire includes information on the time-use of workers. While it is not possible to use the information on hours and months worked due to imprecisely recorded data, we use months worked per year, and take a 12 months working relation to be a full-time employee. If the household or non-household worker worked less than 12 months, we take a fraction, for example 0.5 for a six months work relation, and the enterprise owner is assumed to work full-time. The outcomes are shown in Fig. 10b.

The differences are statistical significant in both figures. The difference for Malawi (1) is statistically significant at the 1 percent, and for Malawi (2) at the 5 percent level. In both figures urban enterprises are more productive than rural enterprises, but the differences diminish compared to the measure used in Section 5.

Fig. 11 uses the same productivity measures as Fig. 10 accounting for the gender of the enterprise owner. Compared to the kernel density estimates in Fig. 7, the differences between male and female enterprise owners almost disappear. The differences are not significant for Malawi (1) and significant at a 10 percent level for Malawi (2), where male enterprise owners report a higher productivity level compared to female enterprise owners.

## Footnotes

1 In this paper non-farm entrepreneurship is described as any kind of business activity in the non-farm economy that is undertaken by the active rural population. Non-farm enterprises are business ventures started and managed by households outside of agriculture. They typically include many heterogeneous activities such as food-processing, trade and sales, services, as well as construction and transportation activities (Wiggens and Hazell, 2011). Although referred to as non-farm, many of these enterprises are linked to agriculture and can be located on a farm, for example food processing or veterinary services (Rijkers and Costa, 2012).

2 For additional information on the LSMS-ISA, see also the World Bank’s website www.worldbank.org/lsms-isa

3 See Table 7 in Appendix A for the prevalence of non-farm entrepreneurship in urban areas.

4 There are only sufficient responses for Ethiopia in the LSMS-ISA data.

5 Household income is calculated closely following the definition by the International Labour Organization (ILO), where household income consists of all receipts whether monetary or in kind that are received by the household or individual household members at annual or more frequent intervals. It excludes windfall gains and other irregular or one-time receipts. The household income calculated in the RIGA data is based on different sources of income: wage, non-wage, dependent and self-employment, that a household can earn over a well-defined reference period. See also Carletto et al. (2007).

6 The data from Niger has to be read with care, since the categories are not equal to the ones for Ethiopia and Malawi. We therefore group different activities, and allocate approximate business activities to match the categories in Ethiopia and Malawi.

7 See Appendix A for summary statistics and a detailed definition of the variables.

8 Additionally we run this probit model with the aggregate sample, using binary variables to account for the different countries. Overall rural households are 17 percentage points less likely to operate an enterprise compared to urban households. We also note a significant country-level heterogeneity in the probability of operating an enterprise, which is reflected in Table 1. The results are available upon request.

9 The variables included in this analysis also contain various polynomials, such as the square of age, number of adults, and distance to population center. However, we only report the average marginal effects.

10 The data does not cover urban areas in Ethiopia. Instead the differentiation is made between rural areas and small towns. We assume that the smaller difference in productivity dispersal stems from this data limitation.

11 The figures are reported in Appendix B.

12 See Appendix A for summary statistics and a detailed definition of the variables.

13 If the enterprise employs additional workers, the number of workers are 2.81 for urban and 2.07 for rural enterprises. The large majority of enterprises, however, do not employ any additional workers: 72.71 percent of all enterprise in urban and 85.29 percent in rural areas are exclusively operated by the enterprise owner without further workers (household or non-household members).

14 All shares are weighted.
